# Altered neuronal excitability underlies impaired hippocampal function in an animal model of psychosis

**DOI:** 10.3389/fnbeh.2015.00117

**Published:** 2015-05-20

**Authors:** Thomas Grüter, Valentina Wiescholleck, Valentyna Dubovyk, Verena Aliane, Denise Manahan-Vaughan

**Affiliations:** ^1^Medical Faculty, Department of Neurophysiology, Ruhr University BochumBochum, Germany; ^2^International Graduate School of Neuroscience, Ruhr University BochumBochum, Germany

**Keywords:** hippocampus, schizophrenia, NMDA receptor hypofunction, MK801, GABA, *in vivo*

## Abstract

Psychosis is accompanied by severe attentional deficits, and impairments in associational-memory processing and sensory information processing that are ascribed to dysfunctions in prefrontal and hippocampal function. Disruptions of glutamatergic signaling may underlie these alterations: Antagonism of the N-methyl-D-aspartate receptor (NMDAR) results in similar molecular, cellular, cognitive and behavioral changes in rodents and/or humans as those that occur in psychosis, raising the question as to whether changes in glutamatergic transmission may be intrinsic to the pathophysiology of the disease. In an animal model of psychosis that comprises treatment with the irreversible NMDAR-antagonist, MK801, we explored the cellular mechanisms that may underlie hippocampal dysfunction in psychosis. MK801-treatment resulted in a profound loss of hippocampal LTP that was evident 4 weeks after treatment. Whereas neuronal expression of the immediate early gene, Arc, was enhanced in the hippocampus by spatial learning in controls, MK801-treated animals failed to show activity-dependent increases in Arc expression. By contrast, a significant increase in basal Arc expression in the absence of learning was evident compared to controls. Paired-pulse (PP) facilitation was increased at the 40 ms interval indicating that NMDAR and/or fast GABAergic-mediated neurotransmission was disrupted. In line with this, MK801-treatment resulted in a significant decrease in GABA(A), and increase in GABA(B)-receptor-expression in PFC, along with a significant increase of GABA(B)- and NMDAR-GluN2B expression in the dentate gyrus. NMDAR-GluN1 or GluN2A subunit expression was unchanged. These data suggest that in psychosis, deficits in hippocampus-dependent memory may be caused by a loss of hippocampal LTP that arises through enhanced hippocampal neuronal excitability, altered GluN2B and GABA receptor expression and an uncoupling of the hippocampus-prefrontal cortex circuitry.

## Introduction

Changes in neurotransmission, mediated by the glutamatergic, dopaminergic and GABAergic systems may underlie pathophysiological changes in the brain that lead to psychosis (Laruelle et al., 1996; Kristiansen et al., 2006; Abi-Dargham et al., 2012; Stan and Lewis, 2012). However, dopamine receptor ligands treat only a subset of the symptoms (Chue and Lalonde, 2014), and pharmacological treatment to regulate neurotransmission through GABA(A) receptors, fails to ameliorate symptoms (Dold et al., 2013). In psychosis, hypofunction of glutamatergic N-methyl-D-aspartate receptors (NMDAR) is believed to occur (Gunduz-Bruce, 2009). Furthermore, NMDAR-antagonists trigger psychotic episodes in healthy adults and exacerbate symptoms in psychosis patients (Lahti et al., 2001). An interplay with the dopaminergic and GABAergic systems is likely (Healy and Meador-Woodruff, 1996; Cochran et al., 2003; Hashimoto et al., 2003; Kinney et al., 2006). Moreover, a reciprocal regulation of NMDAR and GABA-receptor function may lie at the core of psychosis (Gordon, 2010; Wiescholleck and Manahan-Vaughan, 2013a) and mediate dopaminergic dysfunction in the disease (Schwartz et al., 2012).

Early clinical intervention in psychosis greatly increases the chances of improved clinical outcome (Álvarez-Jiménez et al., 2012). Thus, an understanding of the mechanisms that accompany the early stages of the disease is key to the development of effective therapeutic strategies. Notwithstanding their limitations, animal models of NMDAR-hypofunction emulate many of the symptoms of psychosis seen in humans (Neill et al., 2010; Wiescholleck and Manahan-Vaughan, 2013a). Prolonged treatment of rats with the irreversible NMDAR-antagonist, MK801, emulates the chronic and advanced phase of psychosis (Wiescholleck and Manahan-Vaughan, 2013a). Furthermore, following acute, one-off systemic treatment, animals exhibit symptoms of first-episode psychosis (Wöhrl et al., 2007; Manahan-Vaughan et al., 2008a,b), but also show long-lasting cognitive deficits (Manahan-Vaughan et al., 2008a,b; Wiescholleck and Manahan-Vaughan, 2012, 2013b; Galizio et al., 2013; Lobellova et al., 2013). These changes emulate deficits in hippocampus-dependent memory, behavioral flexibility, attention, and working memory that are known to occur in psychosis patients (Rajji et al., 2009).

Hippocampal synaptic plasticity may underlie information storage (Kemp and Manahan-Vaughan, 2007), and cognitive processes such as the learning of spatial scenes, or contextual fear memory are tightly associated with LTP (Straube et al., 2003; Whitlock et al., 2006; Kemp and Manahan-Vaughan, 2007). LTP-like plasticity and learning are deficient in schizophrenic patients after transcranial magnetic, or direct current stimulation (Frantseva et al., 2008; Hasan et al., 2011), whereby both induce synaptic plasticity in the cortex of healthy adults (Stefan et al., 2000; Liebetanz et al., 2002). Strikingly, in close parallel to these observations in humans, a profound loss of hippocampal LTP that is persistent (>months), and is accompanied by a significant loss of hippocampus-dependent memory occurs in the MK801-animal model of psychosis (Manahan-Vaughan et al., 2008a,b; Wiescholleck and Manahan-Vaughan, 2012, 2013b). This suggests that the hippocampus may be at the center of cognitive changes that occur in psychosis and raises the question as to the mechanisms that underlie these alterations.

NMDAR-hypofunction reduces inhibitory GABAergic neurotransmission (Schwartz et al., 2012). We postulated that the loss of LTP and associated changes in memory function seen in humans and rodents could arise from changes in glutamatergic neurotransmission and increased neuronal excitability in the hippocampus. To clarify this, we examined the mechanisms that underlie LTP impairments and loss of hippocampus-dependent memory (Manahan-Vaughan et al., 2008a,b; Wiescholleck and Manahan-Vaughan, 2012, 2013b) that occur following an acute systemic treatment with the NMDAR-antagonist, MK801 (Wiescholleck and Manahan-Vaughan, 2013a). We observed that a chronic elevation in neuronal excitability occurred in the hippocampus that was accompanied by a disruption of inhibitory control in the prefrontal cortex. These changes were mediated by alterations in expression of the NMDAR subunit GluN2B in the hippocampus, along with changes in GABA(A) and GABA(B) expression in both the hippocampus and prefrontal cortex. We propose that in psychosis, deficits in hippocampus-dependent memory are caused by a loss of hippocampal LTP that arises through enhanced hippocampal neuronal excitability, altered glutamate and GABA receptor expression, and an uncoupling of the hippocampus-prefrontal cortex circuitry.

## Materials and Methods

### Animals

The present study was carried out in accordance with the European Communities Council Directive of September 22nd 2010 (2010/63/EEC) for care of laboratory animals and after approval of the local government ethics committee (Bezirksamt, Arnsberg). Male Wistar rats (7–8 weeks, Charles River, Germany) were housed individually in a temperature- and humidity-controlled vivarium with a constant 12-h light-dark cycle (lights on from 6 a.m. to 6 p.m.) with *ad libitum* food and water access. All surgical procedures and experiments were conducted during the day.

### Compounds and Drug Treatment

The NMDAR antagonist [+]-5-methyl-10, 11-dihydro-5H-dibenzo-[a, d]-cyclohepten-5, 10-imine maleate (MK801, Tocris, Germany) was dissolved in 0.9% physiological saline. MK801 (5 mg/kg) or vehicle (10 ml/kg) were injected intraperitoneally (i.p.) 7 days before commencement of experiments. The concentration of MK801 was chosen in accordance with previous studies conducted by our group (Wöhrl et al., 2007; Manahan-Vaughan et al., 2008a,b), in which the same dose proved to be effective in inducing long-lasting effects. A single high-dose treatment, as opposed to chronic low-dose treatment, was chosen in order to specifically emulate the very first acute psychosis-related experience (Wiescholleck and Manahan-Vaughan, 2013a). Directly after injection, psychosis-like behaviors (locomotion, ataxia and stereotypy) were observed. Twenty-four hours after treatment the animals’ behavior was not different from vehicle injected controls, as described previously (Wöhrl et al., 2007). No differences in locomotion ability, grooming or rearing behavior was observed over multiple days after treatment, in line with our previous findings (Manahan-Vaughan et al., 2008a).

### Electrophysiology Procedures

#### Surgical Implantations

Animals were anesthetized (52 mg/kg pentobarbital via intraperitoneal injection, i.p.) and underwent chronic implantation of electrodes as described previously (Wiescholleck and Manahan-Vaughan, 2013b). Specifically, a monopolar recording electrode (1 mm diameter, 3.1 mm posterior to bregma, 1.9 mm lateral to the midline) was placed in the dentate gyrus granule cell layer and a bipolar stimulating electrode was placed in the medial perforant pathway (1 mm diameter, 6.9 mm posterior to bregma, 4.1 mm lateral to the midline). The animals were allowed between 7 and 10 days to recover from surgery before experiments were conducted. Pre- and post-operative analgesia was implemented using meloxicam (0.2 mg/kg, i.p.) and local administration of xylocaine.

Responses were evoked in freely behaving animals by stimulating at low frequency (0.025 Hz, 0.2 ms stimulus duration, 10,000 Hz sample rate). For each time-point, five evoked responses were averaged. Dentate gyrus population spike (PS) amplitude, as well as field excitatory postsynaptic potential (fEPSP) slope were monitored. Each experiment started with an input-output (i/o) curve (maximal stimulation 900 μA) to determine the stimulus intensity required to elicit a PS that was of 40% of the maximum obtained in the i/o curve. The i/o-curves between vehicle- and MK801-treated animals did not differ at any time-point after treatment. To ensure stability of recordings and to assess basal synaptic transmission, all animals were tested in a baseline experiment first, where only test-pulse stimulation was applied.

LTP was induced by high-frequency stimulation (HFS) (10 bursts of 15 pulses at 200 Hz with 10 s interburst interval) and was recorded 1 week before (pretreatment LTP control) and 4 weeks after MK801- or vehicle-treatment. PS and fEPSP values for pretreatment LTP did not differ significantly in between the two experimental groups. Short-term potentiation (STP) was induced by weak HFS (wHFS, 3 bursts of 15 pulses at 200 Hz with 10 s interburst interval).

Paired-pulse (PP) measurements, as a tool for assessment of general excitability and neurotransmission, were performed by PP stimulation every 40 s with interpulse intervals (IPI) of 20, 25, 40, 50, 100, 300, 500 ms and 1 s. The whole protocol was applied 3 times with an interval of at least 20 min.

At the beginning of each experiment, baseline evoked potentials were assessed by averaging the response to stimulation (five sweeps at 40 s intervals), every 5 min over a period of 60 min. At this point, either HFS or wHFS was given, and three additional measurements at 5 min intervals were taken, followed by recordings at 15 min intervals for 24 h.

#### Histology

At the end of the electrophysiological study, brains were removed and histological verification of electrodes and cannula localization was carried out, as described previously (Goh and Manahan-Vaughan, 2013). Brain sections (16 μm) were stained according to the Nissl method using 1% toluidine blue and then examined using a light microscope. Data from animals, in which an incorrect electrode or cannula localization was found, were excluded from analysis.

### Compartment Analysis of Temporal Activity by Fluorescence *in situ* Hybridization

We examined Arc mRNA expression in rats that received a systemic MK801 injection 4 weeks previously, compared to the Arc mRNA expression in rats that received a systemic vehicle injection. In a separate study, we analyzed Arc mRNA expression that is triggered by novel holeboard learning in rats that were treated with vehicle or MK801. Testing was conducted in a gray perspex box (40 × 40 × 50 cm) that contained a hole (5.5 cm diameter and 4.5 cm deep) in the floor of each corner of the box (Kemp and Manahan-Vaughan, 2008a). Typically, novel exploration of a novel empty holeboard facilitates LTP that lasts for at least 24 h in rats (Kemp and Manahan-Vaughan, 2008a), and is prevented by pharmacological antagonists that interfere with LTP (Kemp and Manahan-Vaughan, 2008b; Lemon and Manahan-Vaughan, 2012). Holeboard learning in the absence of afferent stimulation, is also impaired by the same antagonists (Hagena and Manahan-Vaughan, 2012). All animals were handled for 2 days before experiments. The rats participated in the following behavioral protocol: One day before the experiment, they were habituated for 1 h to a recording chamber. On the experimental day, they were acclimatized to the same chamber again for at least 60 min. Following this, the empty holeboard was inserted. After 6 min of exploration time, starting after the first direct hole exploration, brains were removed. The exploration time of each hole was counted separately.

On each experiment day, the holeboard experiments were performed for the same number of vehicle and MK801 injected rats. However, the animals that did not perform holeboard exploration were sacrificed on a different day than the rats that performed holeboard exploration. Thus, no direct statistical comparison between these two groups (Arc expression in the absence of learning vs. Arc expression following learning) was conducted.

After extraction, the brains were quick-frozen and stored at −80°C. The unfixed brains were cut coronally 20 μm thick on a Cryostat (Leica CM 3050S) at approximately −20°C, mounted directly on superfrost plus slides (Gerhard Menzel GmbH, Braunschweig, Germany) and stored at −80°C until further processing.

Compartment analysis of temporal activity by fluorescence *in situ* hybridization (catFISH) was conducted by adapting the procedure used by Guzowski and Worley (2001). The Arc cDNA was produced by Entelechon (Bad Abbach, Germany) according to the sequence of Lyford et al. (1995). Using the linearized DNA, the antisense RNA probe labeled with fluorescein at the uridine-5’-triphosphate site was created using the *in vitro* transcription kit Ambion MaxiScript Kit (Invitrogen, Carlsberg, USA). The length of the RNA probe was monitored by gel electrophoresis.

Following fixation with 4% paraformaldehyde (PFA), brain slices were deposited in a solution of acetic anhydride (Acros Organics, New Jersey, USA) with diethylpyrocarbonate water (C. Roth, Karlsruhe, Germany) and triethanolamine followed by an acetone/methanol (1:1) solution for 5 min. Afterwards, the slides were set in a humid chamber that was prepared with a 50% deionized formamide solution (Sigma-Aldrich, St.Louis, USA). After covering with 1×-prehybridization buffer (Sigma-Aldrich, St.Louis, USA), they were incubated for 30 min.

For the hybridization step, for each glass slide 1 ng mRNA probe was dissolved in 1 μg 1×-hybridization buffer (Sigma-Aldrich, St.Louis, USA), heated at 90°C for 5 min and placed directly on ice. The slices were incubated with the Arc mRNA in a humidity chamber at 56°C overnight. Then, 10 μg/ml ribonuclease A (from bovine pancreas, Sigma-Aldrich) was added for 15 min at 37°C. To block endogenous peroxidase, the slices were pretreated with 3% H_2_O_2_ for 15 min, and in order to inhibit unspecific binding of proteins, a solution of 10% normal goat serum (n-Goat) was added for 60 min in the humid chamber. Anti-fluorescein-peroxidase (polyclonal sheep Fab-fragment, 11426346910, Roche Diagnostics, Mannheim, Germany) was applied in a dilution of 1:400 in 1% n-Goat for 2 h. After washing 3 times 5 min with tris-buffered saline (TBS)-Tween 20 (Polysorbate 20), a solution of biotinylated tyramine (bT), H_2_O_2_ (0.02%), and TBS was applied (1:1:100) for 30 min to enhance the fluorescence signal. Then, the slices were incubated in a dilution of StrepAvidin Cy5 (Dianova, Hamburg, Germany) 1:3000 with 1% bovine serum albumin (BSA, Sigma Aldrich, St. Louis, USA) in TBS-Tween for 30 min. In order to label the nuclei of the cells, 4’, 6-diamidino-2-phenylindole (DAPI, Invitrogen, Carlsberg, USA) was added in a concentration of 1:10000.

For glutamic acid decarboxylase 2 (GAD67) staining, the slices were incubated in 10% n-Goat and 0.2% Triton X (Tx, Sigma Aldrich, St. Louis, USA) for 90 min on the next day. Afterwards, they were treated with the GAD67 antibody (AB) (1:100, monoclonal mouse AB, MAB-5406, Merck Millipore, Billerica, USA) and 1% n-Goat and 0.2% overnight. On the next day, the slices were incubated with Dylight-488 (1:250, Cy2-labeled goat-anti-mouse AB, 115-485-166, Dianova) and 1% n-Goat and 0.2% Tx and coverslipped with Vectorshield HardSet mounting medium for fluorescence (Vector Laboratories, Burlingame, USA).

As a dilution medium, saline-sodium citrate buffer were used until the anti-fluorescein step, followed by TBS. DAPI and the following procedures were conducted with phosphate buffered saline (PBS) as the dilution medium.

### Immunohistochemistry

All animals were handled for 2 days before experiments. To arrest further brain degradation processes, the rats were perfused with 4% PFA and the extracted brains were stored and cryoprotected in PFA followed by sucrose (30%) for 5 days at 4°C. Serial coronal sections (30 μm thick) were prepared using a freezing microtome. To minimize errors, or variations in the processing of data sets, slices from vehicle and MK801-treated animals were processed together.

In order to reduce unspecific background staining, endogenous peroxidase was blocked by 0.3% H_2_O_2_ and then, endogenous biotin and electrostatic loadings of proteins were reduced by 20% avidin (Avidin-biotin blocking kit, Vector Laboratories, Burlingame, USA). Afterwards, the primary antibodies (AB) were applied in 20% biotin (Avidin-biotin blocking kit) overnight in their corresponding concentration: **GABA(A)** (1:400, monoclonal mouse AB, MAB341, Merck Millipore, Billerica, USA), **GABA(B)** (1:500, monoclonal mouse AB, ab55051, Abcam, Cambridge, UK), **GluN1** (1:400, monoclonal mouse AB, 556308, PharMingen, Becton, Dickinson and Company, Franklin Lakes, USA). **GluN2A** (1:750, polyclonal rabbit AB, sc-9056, Santa Cruz Biotechnology, Santa Cruz, USA), and **GluN2B** (1:200, polyclonal goat AB, sc-1469, Santa Cruz Biotechnology, Santa Cruz, USA), The secondary AB, rat-absorbed biotinylated horse-anti-mouse (BA-2001), goat-anti-rat (BA-1000), and horse-anti-goat antibodies (BA-9500) (all from Vector Laboratories, Burlingame, USA), were added 1:500 for 90 min. After applying the ABC-Elite detection system 1:1000 for 90 min, the reaction was visualized by incubation in 0.05% 3, 3’-Diaminobenzidine-solution (DAB, Sigma-Aldrich, St.Louis, USA) and 0.01% H_2_O_2_ for 10 min.

For staining of GABA(A), GABA(B) and GluN2B the dilution medium contained 10% normal serum (Vector Laboratories, Burlingame, USA) diluted in PBS containing Tx. For staining of GluN1 and GluN2A, 1% BSA in TBS containing Tx was used as the dilution medium.

To enhance the staining of GluN1 and GluN2A, the sections were left in the primary AB solutions for 5 days at 4°C. Following the secondary AB, the sections were enhanced with bT as described by Adams (1992). After mounting on gelatinized slides and dehydration, they were cover-slipped with DePeX (Serva, Heidelberg, Germany).

**Figure 1:**
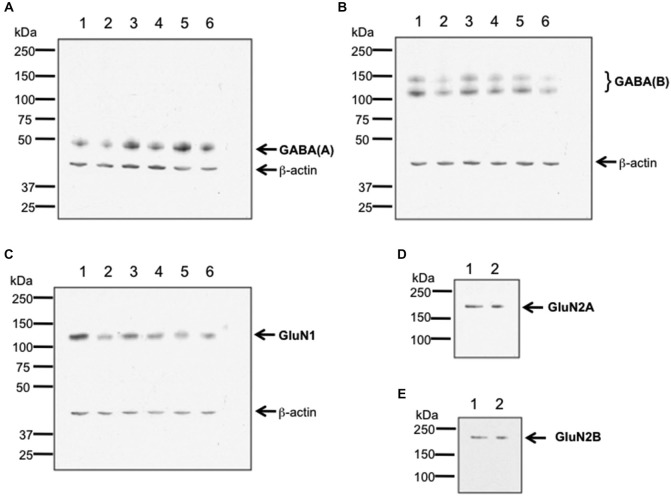
**Verification of antibody specificity. (A–C)** Examples of western blots showing binding specificity of the antibodies used in the immunohistochemistry experiments: the GABA(A) antibody labeled a band of ca. 50 kDa **(A)** corresponding to the target receptor (Olsen and Tobin, 1990). Two bands were labeled by the GABA(B) receptor antibody **(B)**, that correspond to the GABA(B) receptor subtypes 1 and 2 (Kaupmann et al., 1998). The GluN1 antibody **(C)** labeled a ca. 120 kDa band, corresponding to the reported kDa weight of GluN1 (Riou et al., 2012). β-actin (42 kDa) was used as a protein-loading control. Hippocampal tissue was used for assessment. Each lane corresponds to a separate sample. **(D,E)** Examples of immunoblots showing binding specificity of GluN2A **(D)** and GluN2B **(E)**. The GluN2A and GluN2B antibodies labeled ca. 177 and ca. 178 kDa bands, respectively. These bands correspond to the reported kDa weights of the target receptors (Riou et al., 2012). Hippocampal tissue was used for assessment. Each lane corresponds to a separate sample.

To verify antibody specificity, we conducted western blotting of GABA(A), GABA(B) and GluN1 receptors, and used an immunoprecipitation approach to assess GluN2A and GluN2B (Burry, 2000; Figure 1). Hippocampal tissue from 6–8 week old male Wistar rats was used. Hippocampi were homogenized at 4°C in Tris-HCI buffer (20 mM, pH 7.4) containing 10% sucrose. Homogenates were centrifuged at 4°C for 30 min with 14,000 g. Pellets were resuspended in ice-cold Tris-HCl buffer, pH 7.4 that contained cocktail of protease inhibitors (Sample buffer). The total protein concentration of each sample was determined using a Bradford protein assay.

For western blot experiments, 20 μg protein was applied to each gel. Gel electrophoresis was conducted using 8% sodium dodecyl sulfate (SDS) polyacrylamide gels run on a mini-gel apparatus (Pequlab), for 1.5 h at room temperature. The western blot transfer was conducted in cold transfer buffer (wet conditions) using a western blot transfer system (Consort) at 400 V, 250 mA for 1.5 h. Membranes were incubated for 1 h in TBS buffer (100 mM Tris-HCl; 0.9% NaCl, 1% Tween 20, pH 7.4) containing 5% non-fat dry milk to block nonspecific binding sites. Blots were subsequently incubated overnight at 4°C with antibodies that labeled GluN1 (1:750 dilution), Glun2A (1:250 dilution), GluN2B (1:500 dilution), GABA(A) (1:500 dilution), or GABA(B) (1:500 dilution). As a loading control, we used β-actin that was labeled using a monoclonal antibody (1:20000 Sigma, St. Louis, USA).

For immunoprecipitation experiments, a total of 200 μg of protein of tissue lysate was filled up to 400 μl of total volume with sample buffer and incubated with either anti-GluN2A or anti-GluN2B antibody. Immunocomplexes were captured by incubating overnight with protein A Sepharose beads at 4°C. Beads were washed three times in Sample buffer. Immunoprecipitated proteins were eluted with Laemmli buffer 2x and immunoblotted using the indicated primary antibodies (1:250 dilution).

For both western blots and immunoblots, immunoreactivity was revealed using HRP-coupled anti-rabbit or anti-goat secondary antibodies (1:10,000 dilution, GE Healthcare, Freiburg, Germany) and the enhanced ECL detection system (GE Healthcare, Freiburg, Germany). Quantification was done by densitometric analysis using the programs ScanJet (Hewlett Packard, Palo Alto, USA) and ImageJ 1.45S (Wayne Rasband, National Institutes of Health, USA, Java 1.6.0_20 (64 bit)). The lanes in Figure 1 correspond to separate hippocampal samples.

### Brain Slice Assessment

**Figure 2:**
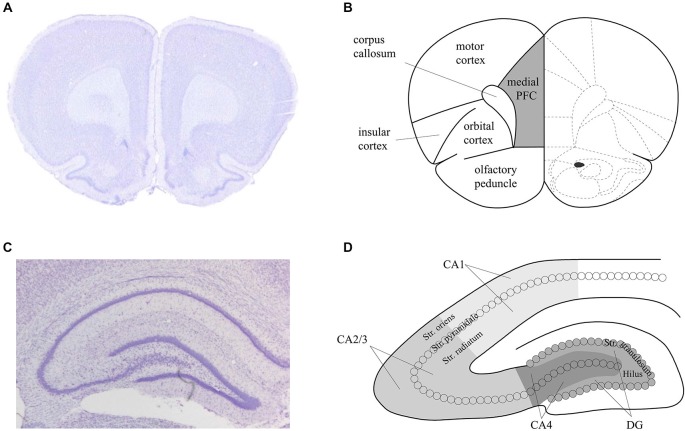
**Schema of the areas analyzed in the prefrontal cortex and hippocampus of the rat.** Nissl stained sections of the prefrontal cortex (PFC) **(A)** and hippocampus **(C)** that correspond to the analysed areas of the medial PFC **(B)** and dorsal hippocampus **(D)** that were assessed in the present study. Figure 1B is based on Paxinos and Watson (1986).

For both the *in situ* hybridization and immunohistochemistry, hippocampal brain areas were assessed at approximately −3.3 mm and −4.3 mm relative to bregma (Figure 2). For the *in situ* hybridization, we analyzed representative and randomly chosen small areas of the DG, the CA3 and the CA1 region. For the immunohistochemistry, we analyzed the whole area of the DG, the CA4, the CA2/3 region and the CA1 region. Additionally, for the immunohistochemistry, prefrontal areas at approximately 3.2 mm and 2.2 mm relative to bregma were used, corresponding to the medial prefrontal cortex. For both techniques, Nissl staining using 1% toluidine blue was made for surveillance of quality and spatial orientation. In addition, negative controls were prepared for supervision of specificity. For this purpose each primary antibody and each secondary antibody was used separately following the staining protocols. No staining could be observed in these negative controls, indicating that the staining observed was specific.

To analyze the amount of receptor expression, we either counted the cells per mm^2^ or we measured the optical density within the subregions of the hippocampus using the software ImageJ (densitometry of the DAB-labeled immunohistochemistry). To correct for differences in the optical density, we subtracted the optical density measured for background staining (within the corpus callosum) from the optical density measured within the subregions of the hippocampus, for each individual slice. This was done to address differences in staining intensity between sections.

### Data Analysis and Statistics

In all electrophysiological experiments, data were expressed as mean % pre-injection values ± standard error of the mean. A mixed ANOVA with the repeated measures factor (TIME) and between-groups factor (GROUP) was used to evaluate differences in plasticity between control experiments and experiments after MK801, or vehicle application.

In the catFISH experiments, MK801 and vehicle-injected groups were compared. For each animal, we labeled three consecutive slices, and z-stacks were obtained from the CA1, CA3 and DG region of one hemisphere of each slice using a Zeiss ApoTome (63× magnification). Only cells were included where the entire nucleus was visible in the z-stack. The percentage of intranuclear Arc-positive cells within the hippocampal regions was analyzed. All GAD67-positive interneurons and glial cells were excluded from the analysis. To control for bias, measurements were carried out blind and additionally spot-checked by two additional individuals. A multifactorial one-way ANOVA using a between-group factor TREATMENT (vehicle vs. MK801) and a between-group factor REGION (PFC, DG, CA4, CA2/3 and CA1 for immunohistochemistry; DG, CA3 and CA1 for *in situ* hybridization) was used to analyze the immunohistochemistry or catFISH data. The catFISH data was furthermore separated according to the behavioral protocol (without holeboard exploration or after holeboard exploration). All data were shown as mean ± standard error of mean. Statistical analysis was performed using the SPSS software (version 19). The level of significance was set at *p* < 0.05.

## Results

### Hippocampal LTP in Behaving Rats is Impaired after MK801-Treatment

**Figure 3:**
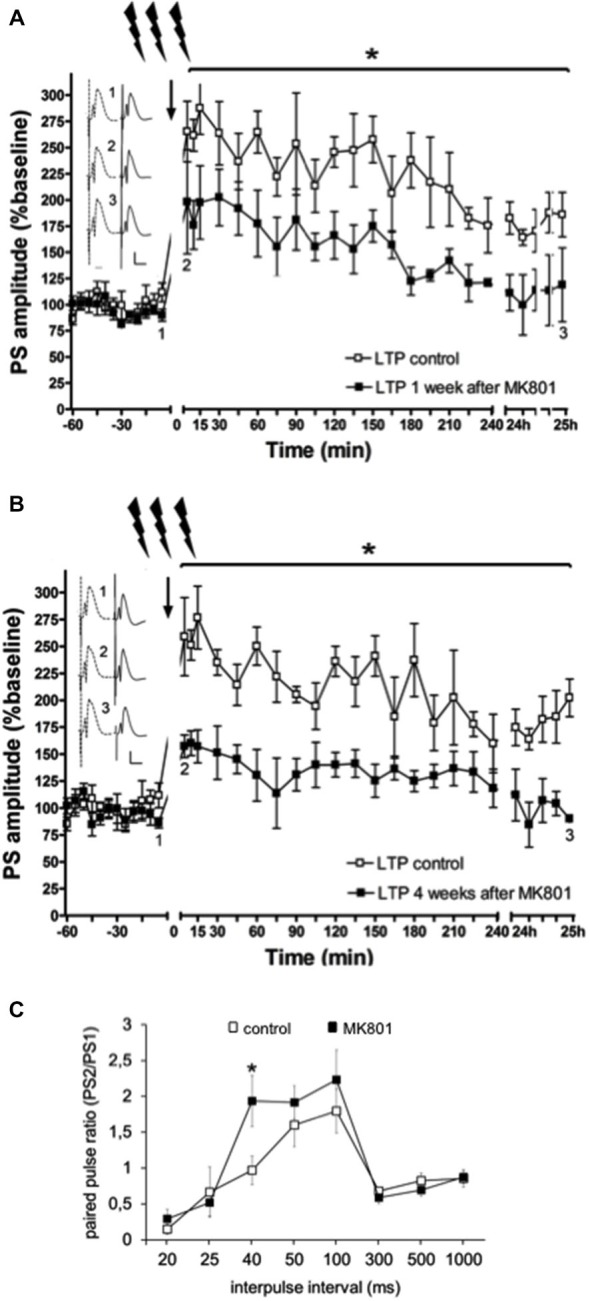
**Electrophysiological responses following MK801-treatment. (A)** MK801-treatment impairs LTP. High frequency stimulation (HFS) elicits robust LTP that persists for over 24 h in animals that were treated 1 week **(A)**, or 4 weeks **(B)** previously with vehicle. In contrast, LTP is profoundly impaired 1 **(A)**, or 4 weeks **(B)** after MK801-treatment. Insets: Analogs (control—dashed line, MK801—black line) were obtained **1**, pre-HFS, **2**, 5 min post-HFS and **3**, 24 h post-HFS. Vertical scale-bar corresponds to 5 mV, horizontal scale-bar to 10 ms. *p < 0.05. **(C)** Paired-pulse (PP) responses are facilitated at the 40 ms interval after MK801-treatment. The graph shows PP responses at the interpulse intervals (IPI) of 20, 25, 40, 50, 100, 300, 500 ms and 1 s. The PP ratio was determined by dividing the amplitude of the second population spike (PS) in mV divided by the amplitude of the first PS (PS2/PS1). PP facilitation was evident at the 40 ms IPI after a single MK801-injection. Responses were unaffected at all other IPIs. *p < 0.05.

In line with previous reports (Manahan-Vaughan et al., 2008a,b; Wiescholleck and Manahan-Vaughan, 2012, 2013b), LTP that was induced by HFS (200 Hz) was significantly impaired in MK801-treated animals 1 and 4 weeks after treatment, compared to animals that received vehicle *F*_(1,6)_ = 36.351, *p* < 0.005, *n* = 4, Figure 3). Control animals expressed robust LTP that lasted for over 24 h, whereas MK801-animals showed an immediate impairment of LTP after HFS. This suggests that a single treatment with MK801 is sufficient to cause prolonged effects on hippocampal function. In line with this, we have observed that hippocampus-dependent learning is also chronically impaired (Manahan-Vaughan et al., 2008a,b; Wiescholleck and Manahan-Vaughan, 2012, 2013b).

### Neuronal Arc Expression is Enhanced in the Hippocampus Following Spatial Learning in Vehicle-Treated Rats. MK801-Treatment Increases Basal Arc Gene Expression in the Hippocampus, but does not Increase Arc Expression after Spatial Learning

The immediate early gene, Arc, is a very useful marker for activity-dependent neuronal activity. Arc gene transcription is rapidly induced by synaptic activity and precipitates in neuronal dendrites of non-GABAergic recently activated neurons (Link et al., 1995; Lyford et al., 1995). Furthermore, Arc is linked to learning and behavior, NMDAR-LTP, homeostatic scaling and structural plasticity (Korb and Finkbeiner, 2011). We therefore used compartment analysis of temporal activity by fluorescence *in situ* hybridization (catFISH) of Arc (Guzowski et al., 1999) to examine whether experience-dependent neuronal activity is altered following learning in the MK801-model.

**Figure 4:**
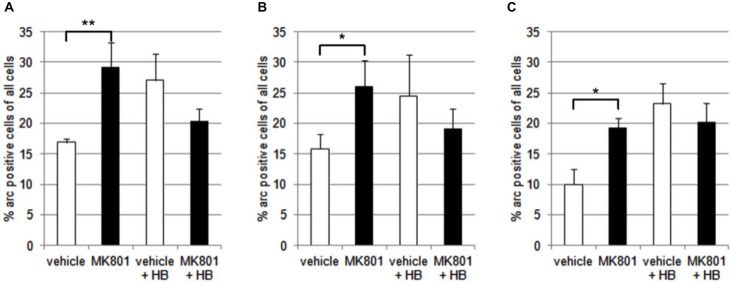
**Overview of Arc expression in naïve animals or following spatial learning**. Bar charts summarize Arc expression in naïve animals that were treated with vehicle or with MK801 4 weeks previously. In the CA1 region **(A)**, the CA3 region **(B)** and dentate gyrus **(C)**, basal Arc expression is significantly increased in MK801-treated animals compared to vehicle-injected controls. In a separate animal cohort, novel spatial exploration resulted in significant elevations of neuronal Arc expression in the hippocampus of vehicle-treated animals compared to their naïve state. This was not the case for MK801-treated animals. Here, no difference was detected in Arc gene expression after novel spatial exploration. *p < 0.05, **p < 0.01.

**Figure 5:**
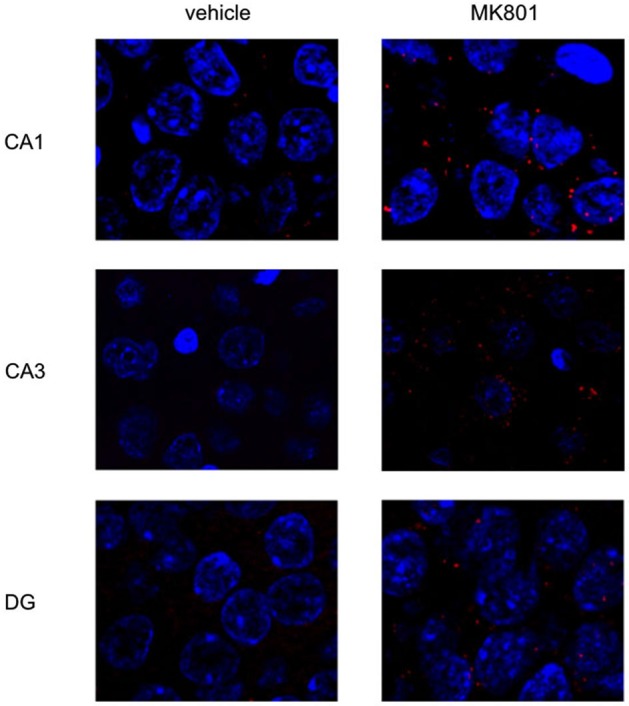
**MK801-treatment alters basal Arc gene expression in the hippocampus**. Spatial learning elicits increases in Arc expression in control, but not MK801-treated animals. The images show Arc expression (red dots) in the CA1 and CA3 regions and dentate gyrus (DG), in naïve animals, after vehicle (left images) or MK801 injection (right images). MK801-treated animals exhibit a significantly higher basal Arc expression in the CA1 and CA3 regions compared to vehicle-treated animals. The nuclei were stained in blue using DAPI. The pictures were taken using a 63× objective.

We first assessed basal Arc gene expression in MK801-treated animals compared to vehicle-injected controls (Figures 4A–C, 5). A multifactorial ANOVA showed, for the group without holeboard exploration, an effect for the factor TREATMENT (*F*_(1,24)_ = 20.61, *p* < 0.001) and the factor REGION (*F*_(2,24)_ = 4.69, *p* < 0.05). No interaction effect TREATMENT * REGION was observed (*F*_(2,24)_ = 0.15, *p* = 0.86). The *post hoc* test showed that 4 weeks after injection, MK801-treated animals (*n* = 5) exhibited significantly higher basal Arc expression in the CA1 region (*p* < 0.01, Figure 4A), the CA3 region (*p* < 0.03, Figure 4B) and the dentate gyrus (*p* < 0.05, Figure 4C) in the MK801-treated animals compared to vehicle-injected controls (*n* = 5). These findings suggest that hippocampal neurons of MK801-treated animals have lower excitability thresholds and may engage in redundant information encoding. Psychosis is characterized by an excessive processing of irrelevant stimuli as well as memory deficits (Anticevic and Corlett, 2012). We therefore asked the question if a learning event, that is known to promote the expression of LTP and result in long-term memory (Kemp and Manahan-Vaughan, 2004) results in increased Arc expression in MK801-animals.

We exposed the animals to a novel holeboard and then examined Arc-related expression in hippocampal neurons. After spatial learning the MK801 treatment did not result in increased Arc expression compared to vehicle injected animals (Figures 4A–C, 5): A multifactorial ANOVA did not reveal an effect for the factor TREATMENT (*F*_(1,24)_ = 2.30, *p* = 0.14), the factor REGION (*F*_(2,24)_ = 0.16, *p* = 0.85) or the interaction TREATMENT * REGION (*F*_(2,24)_ = 0.11, *p* = 0.90).

To rule out that the differences in basal Arc expression observed can be explained by the behavior of the rats, we compared the rats’ activity during exploration of the empty holeboard. We measured the time that each rat spent exploring the different holes, i.e., dipping their heads into one of the holes, within a 6 min period (not shown). The average exploration time of vehicle-treated animals (*n* = 5) was 10.57 ± 1.9 s, and of MK801-treated animals (*n* = 5) was 13.21 ± 1.9 s. No significant difference was evident between the groups (*t*-test: *p* > 0.05).

These findings suggest that the aberrantly enhanced expression of Arc in MK801-treated animals reflects intrinsic elevations in neuronal excitability thresholds that impair signal-to-noise ratios to such an extent, that a novel learning event cannot be effectively encoded. To clarify this, we examined PP responses.

### Paired-Pulse Facilitation is Increased at the 40 ms Interval after MK801-Treatment

Neurotransmission was examined in control animals (*n* = 12) and in animals treated with MK801 (*n* = 14), by stimulating the perforant path with pulse pairs, of constant stimulus intensity, at inter-pulse intervals of 20, 25, 40, 50, 100, 300, 500 and 1000 ms. We found that MK801-treated animals (*n* = 5) showed a PP facilitation at the 40 ms interval compared to control rats (*F*_(1,24)_ = 5.156, *p* < 0.05, *n* = 5) Figure 3C). At all other inter-pulse intervals, there were no differences in PP response between control animals and those that were treated with MK801 prior to testing (20 ms: *F*_(1,24)_ = 0.940, n.s.; 25 ms: *F*_(1,24)_ = 0.156, n.s.; **40 ms**: *F*_(1,24)_ = 5.156, ***p* < 0.05**; 50 ms. *F*_(1,24)_ = 0.734, n.s.; 100 ms: *F*_(1,24)_ = 0.654, n.s.; 300 ms: *F*_(1,24)_ = 0.816, n.s.; 500 ms*: F*_(1,24)_ = 0.951, n.s.; 1000 ms: *F*_(1,24)_ = 0.334, n.s).

This specific interval reflects neurotransmission mediated by NMDAR and GABA(B) receptors, and suggests that neurotransmission is enhanced at perforant path-dentate gyrus synapses of MK801-treated animals. Inhibitory tonus is regulated by GABA(A) and GABA(B) receptors (Mann et al., 2009). Thus, we explored whether expression of these receptors is altered after MK801-treatment. Given its role in memory and its proposed dysfunction in psychosis, we also examined expression in the prefrontal cortex.

### GABA(A) Expression is Decreased in the Prefrontal Cortex, whereas GABA(B) Expression is Increased in Prefrontal Cortex and Dentate Gyrus after MK801-Treatment

**Figure 6:**
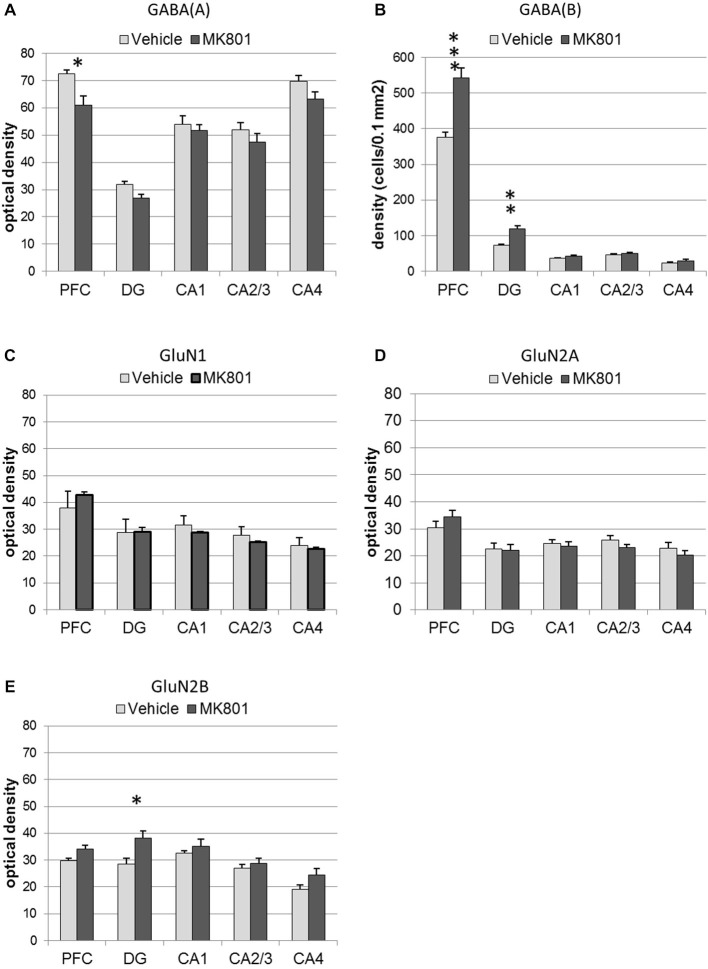
**MK801-treatment alters GABA receptor and N-methyl-D-aspartate receptor (NMDAR) subunit expression in the hippocampus and prefrontal cortex**. Bar charts represent receptor expression in the hippocampal subregions (CA1, CA2/3, CA4, dentate gyrus:DG), and in the prefrontal cortex (PFC) 4 weeks after MK801-treatment (derived from densitometry of the DAB-labeled immunohistochemistry). In MK801-treated animals, **(A)** GABA(A) expression was reduced in the prefrontal cortex but unchanged in the hippocampus, whereas GABA(B) expression **(B)** was significantly increased in both the dentate gyrus and prefrontal cortex compared to vehicle-treated controls. Whereas GluN1 **(C)** and GluN2A **(D)** expression was unaffected by MK801-treatment, GluN2B expression **(E)** was significantly increased in the dentate gyrus compared to vehicle-treated controls *p < 0.05.

**Figure 7:**
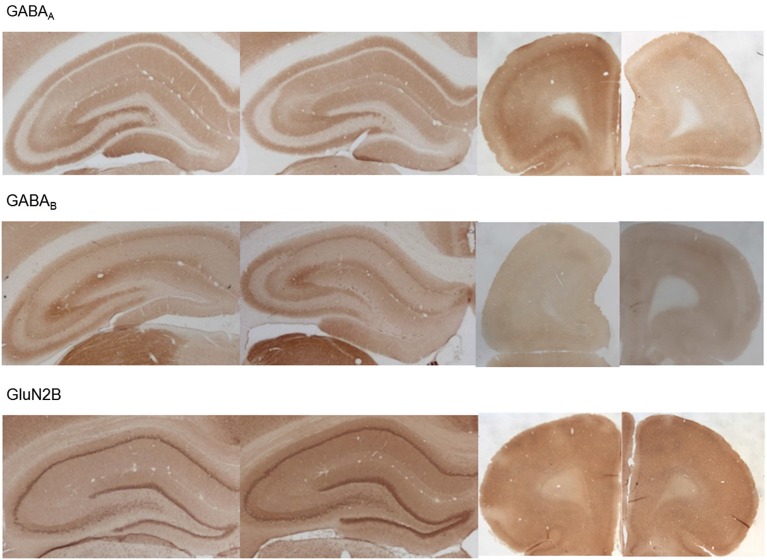
**Photomicrographs of expression of GABA(A), GABA(B) and GluN2B in the hippocampus and prefrontal cortex**. Photomicrographs show examples of receptor expression in the hippocampus (left panels) and prefrontal cortex (PFC) (right panels) 4 weeks after MK801-treatment. In each case, the left panel for the respective subregion shows the control examples. Examples for GABA(A), GABA(B), and GluN2B are shown. Scale bar, hippocampus 1 mm, PFC 5 mm. The examples highlight the decrease of GABA(A)-receptor expression in the prefrontal cortex, the increase of GABA(B)-receptor expression in the dentate gyrus and prefrontal cortex and the increase of GluN2B expression in the dentate gyrus 4 weeks after MK801-treatment.

GABA(A) expression was significantly decreased 4 weeks after MK801 treatment (*n* = 5) compared to vehicle-treated controls (*n* = 5, Figures 6, 7) (multifactorial ANOVA: TREATMENT *F*_(1,40)_ = 14.92, *p* < 0.001, REGION *F*_(4,40)_ = 80.48, *p* < 0.001). Furthermore, a Duncan’s *post hoc* test revealed that the prefrontal cortex was specifically affected (vehicle: 72.50 ± 1.47 vs. MK801: 61.07 ± 3.32; *p* < 0.01), whereas no significant changes were evident in the hippocampal subfields (Figures 5, 6).

GABA(B) expression was significantly increased in MK801-treated animals (*n* = 5) compared to controls (*n* = 5, Figures 6, 7) (multifactorial ANOVA: TREATMENT *F*_(1,40)_ = 42.586, *p* < 0.001, REGION *F*_(4,40)_ = 555.14, *p* < 0.001, interaction TREATMENT × REGION *F*_(4,40)_ = 20.33, *p* < 0.001). In this case, the increases in expression occur in both the prefrontal cortex (Duncan’s *post hoc* test, vehicle: 375.25 ± 14.6 vs. MK801: 542.5 ± 28.79, *p* < 0.001) and the dentate gyrus (Duncan’s *post hoc* test, vehicle: 73.11 ± 3.49 vs. MK801: 118.37 ± 9.99, *p* < 0.01) (Figures 6, 7).

These findings suggest that the alterations in neuronal excitability, and dysfunctional information processing that we detected in MK801-treated animals results from alterations in GABA receptor expression in both the hippocampus and prefrontal cortex.

### GluN2B, but not GluN2A or GluN1 Expression is Increased in the Dentate Gyrus 4 Weeks after MK801 Treatment

With respect to the expression of the NMDAR subunits, GluN1 and GluN2A, no statistically significant differences between vehicle and MK801-treatment could be observed in the prefrontal cortex or the different hippocampal subregions 4 weeks after treatment with either MK801 or vehicle (multifactorial ANOVA, n.s., Figures 6, 7). However, GluN2B expression was significantly increased (multifactorial ANOVA, factor TREATMENT *F*_(1,40)_ = 14.259, *p* < 0.001, factor REGION *F*_(4,40)_ = 13.55, *p* < 0.001, Figures 6, 7). These increases were specific to the dentate gyrus (Duncan’s *post hoc* test, vehicle: 28.60 ± 2.11 vs. MK801: 38.13 ± 2.61, *p* < 0.01).

## Discussion

The results of this study show that deficits in hippocampal LTP in the MK801-animal-model of psychosis are associated with elevations in neuronal excitability that are accompanied by changes in GABA-receptor expression in the hippocampus and prefrontal cortex, along with increased GluN2B-expression in the dentate gyrus. We propose that neuronal hyperexcitability in the hippocampus, coupled with an imbalance of GABAergic inhibitory control in the prefrontal cortex, results in an uncoupling of hippocampus-prefrontal cortex communication, and comprises a key aspect of hippocampal and memory dysfunction in psychosis.

Acute MK801-treatment results in transient behavioral changes that emulate a psychotic episode (Wöhrl et al., 2007; Manahan-Vaughan et al., 2008a). A severe LTP impairment is triggered in the dentate gyrus, that doesn’t recover with time (Wiescholleck and Manahan-Vaughan, 2013b). In the present study, we observed that 4 weeks after MK801-treatment, LTP was still impaired. There are two possible explanations for this deficit: irreversible NMDAR-antagonism chronically disables NMDAR-dependent LTP, or neuronal excitability becomes persistently elevated, such that a further potentiation of synaptic efficacy by LTP is no longer possible. In the dentate gyrus, LTP is not exclusively NMDAR-dependent (Manahan-Vaughan et al., 1998), thus perpetual NMDAR-knock-down may not necessarily result in a complete failure of LTP. In contrast, it has been demonstrated that pushing synaptic potentiation to its upper limits by repeated afferent tetanisation, not only disables the ability of the affected hippocampal synapses to express LTP, but it also prevents new spatial learning (Moser et al., 1998; Moser and Moser, 1999). Given the fact that psychosis patients exhibit a loss of sensory (Javitt, 2009) and sensorimotor gating (Braff et al., 1978) and higher resting BOLD responses in multiple cortical areas (Medoff et al., 2001), we postulated that excitability levels may *increase* in the hippocampus following MK801-treatment, and explored if activity-dependent neuronal activity becomes altered.

Arc is expressed in an activity-dependent manner in response to hippocampus-dependent learning (Guzowski et al., 1999). It is transcribed at a low level under basal (naïve) conditions (Guzowski et al., 2001; Korb and Finkbeiner, 2011). Strikingly, we observed that basal Arc expression was significantly higher in MK801-treated animals compared to controls. In other words, neuronal activation is elevated in MK801-treated animals *even in the absence* of hippocampus-dependent learning. This suggests that the hippocampi of MK801-treated rats may be encoding stimuli and experiences that would normally be dismissed as irrelevant by healthy rats. This is an interesting possibility, as it is known that psychotic patients exhibit excessive processing of irrelevant stimuli that result on the one hand, in cognitive misrepresentations, and on the other hand, in memory deficits (Anticevic and Corlett, 2012). We therefore asked the question if novel spatial learning, that is known to cause a transient and activity-dependent elevation of Arc expression hippocampal neurons, and strengthens LTP (Kemp and Manahan-Vaughan, 2007), can trigger additional Arc expression in MK801-treated animals, that occurs in addition to the already enhanced Arc expression. Strikingly MK801-animals failed to show an increase compared to control animals after the same learning event. This suggests that the elevated levels of basal Arc expression interfere with the encoding of novel spatial experience. We postulate that this aberrantly enhanced expression of Arc in MK801-treated animals reflects intrinsic elevations in neuronal excitability thresholds that impair signal-to-noise ratios to such an extent, that a novel learning event cannot be effectively encoded.

In line with this, paired-pulse (PP) responses were enhanced at the 40 ms interval. In the PP paradigm, two stimuli are delivered with different intervals in the range of 20–1000 ms. The population spike (PS), which reflects the number and synchrony of granule cell discharges (Andersen et al., 1971), exhibits a depression of the 2nd PS at PP-intervals of 20–40 ms, followed by facilitation at PP-intervals of 40–200 ms, and depression at longer intervals. At PP-intervals of 20–40 ms, the PS-depression most likely occurs due to GABA(A)-activation, which in turn leads to an increase in chloride conductance (Albertson and Joy, 1987). At 40–200 ms stimulus intervals, GABA(B)-autoreceptors are active, thereby inhibiting GABA release. At the same time NMDAR are activated (McNaughton, 1982; Albertson and Joy, 1987). The enhanced facilitation we saw at the 40 ms PP-interval may thus relate to a disinhibition of principle cells. This could be triggered by changes in expression of GABA(B) -receptors, or of NMDAR. To explore this we examined the expression and distribution of GABA(A) and GABA(B)-receptors, as well as the GluN1, GluN2A and GluN2B subunits of the NMDAR in the hippocampus.

After MK801-treatment, GABA(B)-receptor and GluN2B expression was enhanced in the dentate gyrus. The CA-subfields of the hippocampus were unaffected. This suggests that increases in neuronal excitability in the dentate gyrus underlie the deficits in LTP we observed. It also explains why PP-responses were increased at the 40 ms interval. GABA(B)-receptors regulate basal inhibitory tonus (Mann et al., 2009), but in the dentate gyrus, they play a very important role in *stimulating* excitability of granule cells, by reducing synaptic inhibition that is mediated by hilar interneurons (Burgard and Sarvey, 1991; Mott and Lewis, 1991; Mott et al., 1993). This serves to promote transfer of incoming information from the entorhinal cortex, via the perforant path, to the hippocampus. Thus, an enhancement of GABA(B)-receptor expression in the dentate gyrus may not only result in neuronal hyperexcitability, it could also result in the processing of irrelevant information by the hippocampus, due to a weakening of gate control of entorhinal inputs. This alteration will be compounded by the increase in GluN2B expression that was specific to the dentate gyrus. GluN2B-containing NMDAR require intense postsynaptic depolarization to remove the voltage-dependent Mg^2+^-block of the NMDAR and result in channel opening (Köhr et al., 2003; Erreger et al., 2005; Berberich et al., 2007), but when activated, they support twice as much charge-transfer (as GluN2A-containing NMDAR), deactivate slower and support a greater Ca^2+^-influx per unit of current (Vicini et al., 1998; Sobczyk et al., 2005). An increase in GluN2B-expression will increase the ease with which incoming stimuli can result in LTP, but if this occurs on a background of enhanced neuronal excitability, it will not necessarily result in persistent or appropriate long-term encoding (Moser and Moser, 1999). Taken together, the enhanced expression of GABA(B)-receptors and GluN2B comprise a highly potent partnership, that can not only be expected to raise elevate neuronal excitability in the dentate gyrus, but mediate redundant synaptic information encoding, impaired LTP, and excessive encoding of experiences that would normally be ignored by the hippocampus. The alterations in dentate gyrus information processing can also be expected to impair information processing in the CA-subfields of the hippocampus (Bikbaev et al., 2008). In line with this, activity-dependent Arc expression was elevated throughout the hippocampus in MK801-treated animals, although increased expression of GABA(B) and GluN2B was localized to the dentate gyrus.

Both hypoactivation and hyperactivation of the prefrontal cortex may contribute to psychosis, and both, when mediated by changes in GABAergic inhibition, lead to attentional deficits (Pezze et al., 2014). Although it is unclear if NMDAR are affected in the prefrontal cortex of psychotic patients (Akbarian et al., 1996; Toro and Deakin, 2005), ketamine-induced psychosis alters metabolic response in this structure (Breier et al., 1997). Thus, we explored if GABA-receptors or GluN-subunits are altered in the prefrontal cortex. Here, we saw no change in GluN-subunits, whereas GABA(A) expression was decreased and GABA(B) expression was increased. These changes can be expected to cause a potent suppression of GABAergic transmission in the prefrontal cortex. Whereas a reduction in GABA(A)-expression under conditions of normal GABA tonus would be expected to result in increased neuronal excitability, the increase in GABA(B)-expression may counteract this to some extent, because basal GABAergic tonus is likely to be reduced (Mann et al., 2009). Nonetheless, it is possible that localized neuronal excitability is increased due to deficits in fast GABAergic transmission, mediated by GABA(A) (Nicoll et al., 1990), leading to aberrant information processing. The increased GABA(B)-expression can be expected to have far-reaching effects. This receptor is important for the modulation of network activity in the prefrontal cortex (Wang et al., 2010), and for the regulation of cortical upstates (Mann et al., 2009) that are essential for the synchronization of long-range cortical activity (Volgushev et al., 2006). Impairments in these processes can be expected to result in attentional and working memory deficits (Major and Tank, 2004), as occur in psychosis (Millan et al., 2012). We propose that the changed expression of GABA-receptors in the prefrontal cortex, could reflect an uncoupling of hippocampus-prefrontal cortex communication: the neuronal hyperexcitability within the hippocampus and associated enhanced output to the prefrontal cortex, will be met by decreased information processing and cortical informational relay that compounds the deficits in hippocampus-dependent long-term memory. These changes may be initiated in the hippocampus: intraperitoneal infusion of MK801 results in an acute enhancement of medial prefrontal cortex potentials that are evoked by hippocampal stimulation (Blot et al., 2015). Thus, alterations in neuronal excitability in the hippocampus may result in adaptive processes in the prefrontal cortex that comprise changed GABA-receptor expression and are aimed to return hippocampus-mediated elevations in excitability back to normal levels.

## Conclusions

Increases in neuronal excitability in the hippocampus of schizophrenics, as indicated by elevations in regional cerebral blood flow, have been reported (Medoff et al., 2001). Furthermore, hypomagnesaemia causes neuronal excitability and leads to psychosis (Morris, 1992). Our data indicate that neuronal hyperexcitabillty in the hippocampus, together with a disruption of inhibitory control in the prefrontal cortex, contribute to the loss of LTP and may comprise a mechanism underlying impairments of learning that occur in this model of first-episode psychosis.

## Author Contributions

Valentyna Dubovyk conducted the antibody specificity experiments (Figure 1), contributed to data analysis and presentation, and the writing of the paper and approved the final version. Valentina Wiescolleck conducted the elctrophysiology experiments described in Figure 3, contributed to data analysis and presentation, and the writing of the paper and approved the final version. Verena Aliane conducted the behavioral and *in situ* hybridization experiments described in Figures 4, 5, contributed to data analysis and presentation, and the writing of the paper and approved the final version. Thomas Grüter conducted the immunohistochemical experiments described in Figures 6, 7, and assisted in the behavioral and *in situ* hybridization experiments described in Figures 4, 5. He also contributed to data analysis and presentation, and the writing of the paper and approved the final version. Denise Manahan-Vaughan designed the experimental approach, created the concept and the hypothesis for the study, supervised all experimental work, analysed the data, and was the main person to write the paper. All authors are accountable for the work described in this paper and worked together to ensure the integrity and reproducibility of the data described.

## Conflict of Interest Statement

The authors declare that the research was conducted in the absence of any commercial or financial relationships that could be construed as a potential conflict of interest.
